# The association of USMLE Step 1 and Step 2 CK scores with residency match specialty and location

**DOI:** 10.1080/10872981.2017.1358579

**Published:** 2017-08-01

**Authors:** Jacqueline L. Gauer, J. Brooks Jackson

**Affiliations:** ^a^ University of Minnesota Medical School, Minneapolis, MN, USA; ^b^ University of Minnesota, Minneapolis, MN, USA

**Keywords:** Licensing exams, USMLE, residency, specialty, career planning

## Abstract

**Background**: For future physicians, residency programs offer necessary extended training in specific medical specialties. Medical schools benefit from an understanding of factors that lead their students to match into certain residency specialties. One such factor, often used during the residency application process, is scores on the USA Medical Licensing Exam (USMLE).

**Objectives**: To determine the relationship between USMLE Step 1 and Step 2 Clinical Knowledge (CK) scores and students’ residency specialty match, and the association between both USMLE scores and state of legal residency (Minnesota) at the time of admission with students staying in-state or leaving the state for residency program.

**Design**: USMLE scores and residency match data were analyzed from five graduating classes of students at the University of Minnesota Medical School (N = 1054).

**Results**: A MANOVA found significant differences (*p *< 0.001) between residency specialties and both USMLE Step 1 and Step 2 CK scores, as well as the combination of the two. Students who matched in Dermatology had the highest mean USMLE scores overall, while students who matched in Family Medicine had the lowest mean scores. Students who went out of state for residency had significantly higher Step 1 scores (*p* = 0.027) than students who stayed in-state for residency, while there was no significant difference between the groups for Step 2 scores. A significant positive association was found between a student who applied as a legal resident of Minnesota and whether the student stayed in Minnesota for their residency program.

**Conclusions**: Residency specialty match was significantly associated with USMLE Step 1 and USMLE Step 2 CK scores, as was staying in-state or leaving the state for residency. Students who were legal residents of the state at the time of application were more likely to stay in-state for residency, regardless of USMLE score.

**Abbreviations:** CK: Clinical knowledge; COMLEX: Comprehensive Osteopathic Medical Licensing Examination; GME: Graduate medical education; NRMP: National Resident Matching Program; UME: Undergraduate medical education; USMLE: United States Medical Licensing Examination

## Introduction

In the USA, medical education is completed through two steps. The first step, undergraduate medical education (UME), is completed after a bachelor’s degree, typically lasts four years, and is historically comprised of a foundational sciences coursework phase and a clinical phase (although the current trend is towards integration of the coursework and clinical experiences). After UME, a student generally proceeds on to graduate medical education (GME), often referred to as ‘residency,’ for further training as a physician. Since medical practice often requires in-depth and specific knowledge and experience, residency programs are focused on training physicians in specific specialties, such as pathology, surgery, or psychiatry. Students are matched with a residency program in their preferred specialty through a competitive process, referred to as ‘The Match,’ facilitated by the National Resident Matching Program (NRMP). One of the major goals of an undergraduate medical education institution is to prepare medical students for a successful residency match.

Every residency program has different criteria for determining whether to accept a candidate into their program. One frequently-used criterion is the applicant’s score on the United States Medical Licensing Examination (USMLE). The USMLE is a three-step examination sponsored by the Federation of State Medical Boards and the National Board of Medical Examiners (NBME) required for physician licensure for all physicians, regardless of training location, to practice in the USA. Typically, for medical students receiving training at Liaison Committee on Medical Education (LCME) accredited institutions in the U.S. and Canada, students take Step 1 of the USMLE at the end of the second year of medical school, and Step 2 Clinical Knowledge (CK) and Clinical Skills (CS) in the fourth year of medical school. Together, Step 1 and Step 2 CK assess a physician’s ability to apply knowledge and concepts to provide safe and effective patient care. Step 1 assesses whether medical students understand and are able to apply important concepts of basic science to medical practice, with special emphasis on principles underlying modes of therapy, health, and disease. Step 2 CK further assesses health promotion and disease prevention and strives to devote attention to incorporating principles of clinical sciences and basic patient-centered skills for safe practices of medicine [1]. Since Step 3 is typically taken well after students have been matched to a residency program, it does not play a role in residency match decisions, and is therefore not analyzed in the current study. The USMLE was not originally intended to be used in selection decisions, but, as it is a rigorous standardized exam that is taken by almost every applicant who participates in the Match, many residency program directors have found it to be a useful measure for comparing candidates from various UME institutions.

The NRMP releases an annual report which includes the medians and interquartile ranges of Step 1 and Step 2 CK scores for the applicants who successfully or unsuccessfully matched in each residency specialty. Since certain residency specialties are more competitive than others, patterns can be found in the USMLE scores of students accepted into each different specialty [2]. These patterns indicate that students may need higher USMLE scores to be successfully matched into certain specialties. Indeed, students are often advised to consider their own USMLE scores when deciding which specialties and programs to rank. In one survey, students applying to residency programs universally regarded Step 1 scores as one of the top academic factors considered by residency program directors when evaluating applicants for residency [3]. Their perceptions are well-founded: in the 2014 national survey of residency program directors across all specialties conducted by the NRMP (N = 1793), the USMLE Step 1 / COMLEX (Comprehensive Osteopathic Medical Licensing Examination) Level 1 score was the most commonly cited factor in selecting applicants to interview, with 94% of respondents indicating that they considered Step 1/ COMLEX Level 1 scores when selecting applicants, while 80% indicated that they considered Step 2 CK / COMLEX Level 2 Cognitive Evaluation (CE) scores. When ranking applicants, 80% of respondents indicated that they considered Step 1 / COMLEX Level 1 scores and 71% Step 2 CK / COMLEX Level 2 CE [4]. In specific specialties, a national survey of neurological residency program directors found that the Step 1 score was rated as the 2nd most important factor overall in the resident selection process [5], and a survey of general surgery program directors found that the Step 1 score was rated as the most important factor in the initial screening of applicants [6].

The use of USMLE scores in the residency program selection process is generally well-supported in the literature, as multiple studies have found USMLE scores to be related to several measures of success in residency programs. Particularly strong associations have been found between USMLE scores and other exam-related measures of success, such as scores on in-training examinations in specialties such as dermatology [7], emergency medicine [8], general surgery [9], and neurology and neurosurgery [10], as well as across specialties in meta-analyses [11,12]. Other positive associations were found between USMLE scores and a resident’s likelihood of successfully passing board examinations [12,13], especially on the first attempt [14,15]. On the other hand, USMLE scores tend to not be as strongly correlated with more subjective outcome measures, such as supervisor and faculty evaluations [10,11,15]. Although some researchers have argued that the validity argument for using USMLE scores in residency program selection is ‘neither structured, coherent, nor evidence-based’ due to the lack of association between USMLE scores and measures of clinical skill acquisition [16], and others warn of the possible existence of racial bias when USMLE scores are used in residency program selection decisions [17], the fact remains that USMLE scores are generally considered, by both applicants and program directors alike, to be an important factor in the residency program selection process.

Medical school leaders can benefit from an enhanced understanding of the factors that affect the residency specialty choices of their students. Previous literature has explored many possible factors that affect specialty choice, including personality and values [18], medical school experiences and financial aid [19], and gender [20]. Research has also shown that residents’ reasons for choosing a specific specialty varies by specialty [20,21]. One factor that has not been as deeply explored is the association between USMLE scores and residency specialty choice. Since USMLE scores are an important part of the residency selection process from the perspectives of both program directors and applicants, and since median USMLE scores vary widely by specialty [2], it follows that a student’s USMLE score might influence the specialty in which they match. One of the goals of the current study was to use statistical analysis to explore this association in a large sample of students, from one institution, across several years. Furthermore, when the NRMP reports match statistics, they report on the specialty students match into for their first year of residency. For many students, the first year is a preliminary residency, and they then continue on into an advanced residency in a different specialty. This advanced residency is the specialty that those students actually intend to practice in. Therefore, the NRMP data may not reflect the actual final career paths of all students. By choosing to use the advanced residency data for our students with multiple residencies, we can more accurately reflect the relationships between exam scores and final specialty career choices.

At a public state institution, such as the institution involved in the current study, medical school leaders may notice a particular physician workforce need in their state, and wonder how to direct students to the needed specialty. In a situation like this, it is also helpful for medical school leaders to understand what might predict the geographic locations where their students go for residency. For example, if a public state university is located in a state that has a deficit of family medicine practitioners, it is incumbent on the leaders of that medical school to select and train future physicians who will both be interested in family medicine and elect to remain in that state. Although a 1982 survey found that geographic region was regarded as the most important factor in choice of specific residency program for seniors from 37 American medical schools [21], the literature on the role of location in specialty program choice is relatively lacking. The current study makes initial attempts at finding patterns in students’ choice of residency program on the dimensions of both specialty and location.

For this study, we analyzed USMLE scores and residency placement data for students from five graduating classes at the University of Minnesota Medical School in order to answer the following research questions: (1) What is the association between USMLE scores and residency specialty? (2) Which specialties have higher mean USMLE scores? (3) Is there a relationship between USMLE score and whether a student stays in-state for residency? (4) Are students who identify as legal residents of the state during the medical school application process more likely to stay in-state for residency?

## Methods

### Institutional approval

Ethical approval for this research was granted by the Institutional Review Board at the University of Minnesota on 19 March 2015. Reference number: 1503E66021.

### Participants

The participants for this study included all students pursuing MD (N = 1049) and MD/PhD (N = 5) degrees at the University of Minnesota who both matriculated between 2007 and 2011 *and* graduated between 2011 and 2015, and who successfully matched into a residency program (total N = 1054). Of the included students, 535 (50.8%) were male and 519 (49.2%) were female. Age at matriculation ranged from 19 to 42 years (M = 23.7 years, SD = 2.5 years). At the University of Minnesota, medical students matriculate at either the Twin Cities campus or the Duluth campus. They complete the first two years of the degree (foundational science courses) at their campus of matriculation, and then all students complete the second two years of the degree (clinical clerkships) through the Twin Cities campus. Of the students in this study, 280 (26.6%) matriculated at the Duluth campus and 774 (73.4%) matriculated at the Twin Cities campus.

### Sources of data

Academic and demographic data, including date of matriculation, date of graduation, and residency match information, were collected from student records held by the Office of Medical Education in the Academic Health Center at the University of Minnesota. Data regarding participants’ age at matriculation, gender, and state of legal residency were retrieved from the University of Minnesota’s access to primary application data through the American Medical College Application Service (AMCAS). For this study, a student’s state of legal residency was determined based on which state they self-identified as their state of legal residency during the AMCAS application process. Data regarding participants’ USMLE Step 1 and Step 2 CK scores were provided to the Medical School by the National Board of Medical Examiners upon students’ completion of each exam.

### Analyses

Using SPSS Statistics v.22 (IBM: USA), we conducted a one-way between-groups multivariate analysis of variance (MANOVA) to investigate residency specialty differences in USMLE scores. The two dependent variables were Step 1 scores and Step 2 CK scores, and the independent variable was residency specialty. We conducted preliminary assumption testing to check for multivariate normality, homogeneity of variance-covariance matrices, and multicollinearity, with no serious violations noted. Due to the wide range of N values in each specialty group (5–205), we chose to use Pillai’s Trace as our test statistic, as it is relatively robust to unequal N values [22]. We also conducted independent-samples t-tests for students who stayed in-state vs. went out-of-state for residency to explore group differences in scores on the USMLE. Finally, we conducted a chi-square test of association to determine whether there was a significant association between whether a student was a legal resident of Minnesota at the time of application to medical school and whether they stayed in-state for residency.

We coded the specialties matched into by our students, following the specialty categories used by the National Resident Matching Program [2]. However, since some specialties were not well-represented in our sample, we combined certain small specialties with related larger specialties. We combined Child Neurology with Neurology, and we also combined several surgical specialties (Neurosurgery, Plastic Surgery, and Vascular Surgery) into a single Neuro/Plastic/Vascular Surgery category. Ultimately, we were left with the following 20 specialties: Anesthesiology (N = 52), Dermatology (N = 17), Emergency Medicine (N = 88), Family Medicine (N = 205), Internal Medicine (N = 199), Internal Medicine/Pediatrics (N = 40), Neuro/Plastic/Vascular Surgery (N = 5), Neurology (N = 25), Obstetrics-Gynecology (N = 50), Ophthalmology (N = 17), Orthopedic Surgery (N = 41), Otolaryngology (N = 15), Pathology (N = 13), Pediatrics (N = 112), Physical Medicine and Rehab (N = 7), Psychiatry (N = 47), Surgery (N = 74), Radiation Oncology (N = 5), Diagnostic Radiology (N = 31), and Urology (N = 11).

For students who were accepted into both a transitional and an advanced residency, we used the specialty and location of the student’s advanced residency in our analyses.

## Results

**Figure 1.**
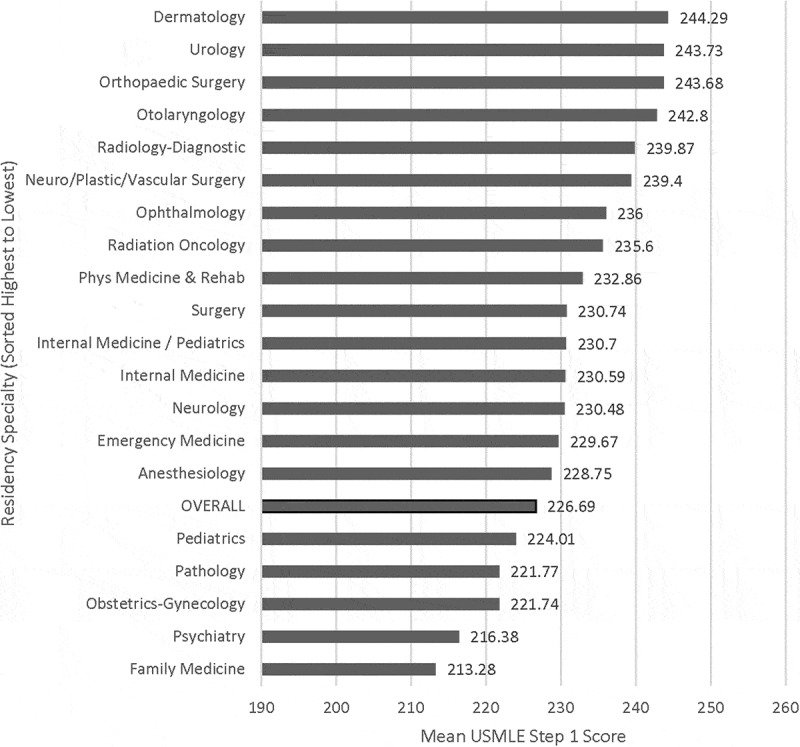
United States Medical Licensing Examination Step 1 mean scores by specialty of residency match for graduates of the University of Minnesota Medical School from 2011 to 2015 (total N = 1054). Overall Step 1 mean score: 226.69.

**Figure 2.**
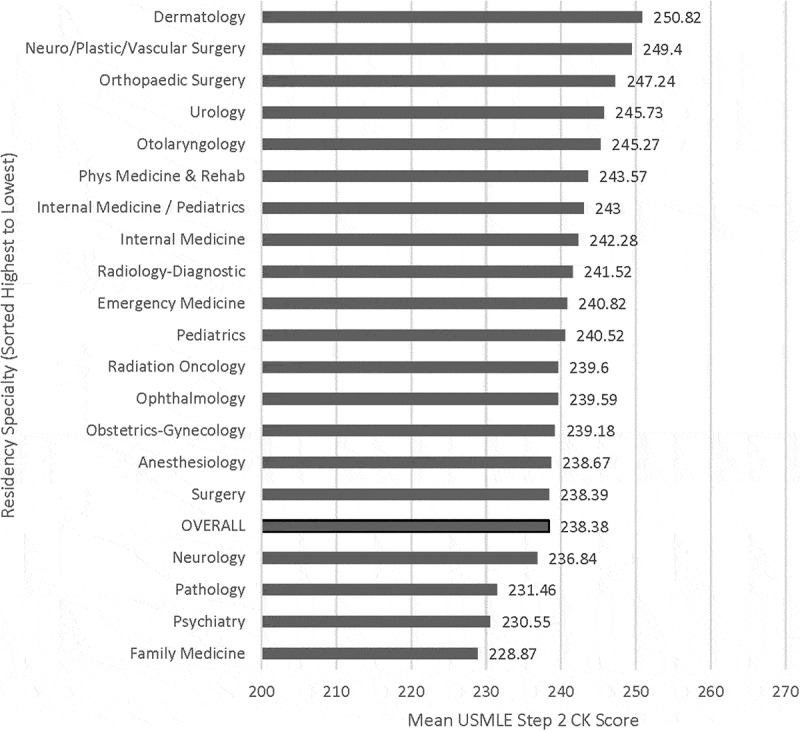
United States Medical Licensing Examination Step 2 Clinical Knowledge (CK) mean scores by specialty of residency match for graduates of the University of Minnesota Medical School from 2011 to 2015 (total N = 1054). Overall Step 2 CK mean score: 238.38.

A one-way MANOVA found a statistically significant difference between residency specialties on the combined dependent variables, F (38, 2068) = 8.307, *p* < 0.001; Pillai’s Trace = 0.265; partial eta squared = 0.132. When the results for the dependent variables were considered separately, both reached statistical significance using a Bonferroni adjusted alpha level of 0.025. For Step 1, F (19, 1034) = 15.427, *p* < 0.001, partial eta squared = 0.221. For Step 2 CK, F (19, 1034) = 7.872, *p* < 0.001, partial eta squared = 0.126. Charts showing the mean Step 1 and Step 2 CK scores by specialty can be found in Figures 1 and 2, respectively. In this dataset, students matching into Dermatology had the highest mean scores, and students matching into Family Medicine had the lowest mean scores, for both Step 1 and Step 2 CK.

When the data are broken down into individual graduating classes, the MANOVA analysis is significant (*p* < 0.001) for each of the five years in the study, as is the univariate ANOVA analysis for Step 1. For Step 2 CK, all years are significant (*p* < 0.01) except for the 2011 graduates.

We conducted independent-samples t-tests to explore whether USMLE Step 1 and USMLE Step 2 CK scores were related to whether students stayed in-state or went out-of-state for residency. We found a significant difference between those who stayed in-state (M = 225.24, SD = 19.084) and those who went out-of-state (M = 227.83, SD = 18.668) at the *p* < 0.05 level on USMLE Step 1 scores (t(1052) = 2.215, *p* = 0.027). However, we did not find a significant difference at the *p *< 0.05 level between those who stayed in-state (M = 237.81, SD = 16.587) and those who went out-of-state (M = 238.83, SD = 16.066) on USMLE Step 2 CK scores (t(1052) = 1.011, *p* = 0.312). These results suggest that, although both Step 1 and Step 2 CK scores were higher on average for students going out-of-state for residency, only Step 1 scores were significantly different.

We also explored whether students who were legal residents of Minnesota were more likely to stay in-state for residency than students who were not legal residents of Minnesota, regardless of USMLE score. Numbers and percentages of residents and non-residents staying or leaving the state for residency can be found in Table 1. Using a chi-square test of association, we found that the percentage of students who went out-of-state for residency did differ significantly by legal residency status, χ^2^(1, N = 1054) = 20.111, *p* < 0.001. Students who were not legal residents of Minnesota at the time of application to the medical school were less likely than those who were legal residents to stay in Minnesota for residency.

**Table 1.** Numbers and percentages of Minnesota (MN) residents and non-residents staying in-state or leaving the state for residency.

Legal residency status	N (percent of total)	Primary residency match in MN	Primary residency match not in MN
*MN* resident	890 (84.4%)	418 (47.0%)	472 (53.0%)
Not *MN* resident	164 (15.6%)	46 (28.0%)	118 (72.0%)
Total	1054	464 (44.0%)	590 (56.0%)

## Discussion

In our dataset of 1054 students, we found significant associations (*p* < 0.001) between both USMLE Step 1 and USMLE Step 2 CK scores and residency specialty, indicating that a relationship exists between exam scores and specialty match. While this is the first study we are aware of that explores these differences statistically, this finding is consistent with previous literature that shows differences in mean exam scores across specialties on the national level. Furthermore, the specialties with the highest (Dermatology) and lowest (Family Medicine) mean USMLE scores in our dataset are consistent with the specialties showing the highest and lowest median scores in the national Match data from the NRMP [2]. It is reasonable to believe that this pattern is maintained at least in part by students with lower scores either not achieving admission to the most competitive programs or self-selecting into specialties with USMLE scores more similar to their own. Conversely, students with high USMLE scores may feel pressured to aim for the more ‘prestigious,’ competitive programs, and students aiming for those competitive programs may dedicate more of their time and energy to studying for the USMLE.

Our results also provide evidence that a relationship exists between USMLE Step 1 scores and students leaving the state for residency. This relationship may be caused by students with higher scores applying to and being accepted by the most prestigious programs, which are not necessarily in-state. Also, the most competitive specialties tend to be smaller, so students may have fewer local choices, and may need to seek programs out-of-state both to find a program they like and to improve their chances of matching. It is important to note that even though this difference was significant, the mean difference was only 2.59 points. Students and institutions may find that difference meaningful or not depending on their context.

Interestingly, our findings did not indicate a relationship between Step 2 CK scores and location of residency. This may be due in part to the timing of when students take Step 2. Many students have not taken Step 2 by the time they apply for the Match, and many programs do not require Step 2 scores in their application process [2]. Therefore, Step 2 scores may be less influential overall in the residency match process.

These findings have implications for public medical schools, such as the University of Minnesota, which are charged with the task of developing future physicians to meet the workforce needs of their state. A large body of literature provides evidence that Medical College Admission Test (MCAT) scores are predictive of USMLE scores [23–25], so if an institution needs to develop, for example, dermatologists, for a state that is lacking in such, the institution may be well-served to select students with high MCAT scores, or to provide extra USMLE study support. Conversely, if a state is in need of more family physicians, a focus on standardized exam scores over other priorities may be unwarranted. The results of the in-state/out-of-state analyses contribute additional nuance to these considerations.

## Limitations

It is important to note that these data reflect the experiences of students at only one medical school. Different medical schools may see different results. However, the national-level data do reflect similar patterns in which residency specialties have higher mean USMLE scores [2].

This study is also limited in its ability to represent all residency specialties equally. Even though the overall sample size of 1054 students is quite large, certain specialties, such as vascular surgery and child neurology, had less than five students, and were therefore combined with larger specialties for the sake of the statistical analysis. For example, only one student in this dataset was matched into a vascular surgery residency program. The USMLE scores of that student would have placed vascular surgery at the top of the lists of specialties ranked by USMLE scores. These nuances are not currently reflected in the results.

Furthermore, even though the smallest specialties were combined, there were still several specialties left with very small sample sizes. This left us with 20 groups, with group sizes ranging from 5 to 205. This makes the interpretation of the MANOVA results more difficult, and also precludes in-depth post-hoc testing. The wide range of group sizes also introduces a concern that the groups would also have differing variances, violating the equality of variances assumption for a MANOVA. However, the standard rule of thumb is that MANOVAs are relatively robust to heterogeneity of variance as long as the largest variance is not more than 4 times the smallest variance. In our sample, the smallest Step 1 variance was 197.82, and the largest variance was 422.80. The smallest Step 2 CK variance was 120.65 and the largest was 351.90. Therefore, our data do not violate the assumption of equality of variance.

One further limitation of this research is that the USMLE was not designed to be used, as it currently is, in the residency match process. Certainly, the use of USMLE scores in this way greatly increases the pressure and anxiety students feel when studying for the licensing exams – instead of simply needing a passing grade, they are competing against other students nationally for the highest score. Several authors have argued that the use of USMLE exams in residency match is not based on evidence of eventual clinical performance [16] and plead for a move to a more holistic approach [26]. However, regardless of whether or not USMLE scores *should* be used in the residency match process, they currently *do* play a large role, and so it is important for medical schools to understand the relationships between USMLE scores and residency choices.

## Conclusion

The results of our study support our hypotheses that there is an association between USMLE scores and residency specialty, and our findings reflect national patterns as to which specialties have the highest and lowest USMLE scores. Students with higher USMLE scores were more likely to match out-of-state, but students who were legal residents of the state upon application to medical school were more likely to remain in-state for residency regardless of USMLE score. These results can help guide decision-makers when determining which students to admit to their institution, and how to best support students in both taking the USMLE and applying for residency.
